# Removal of Large Fibro-Cystic Tumour of Lower Jaw

**Published:** 1872-05

**Authors:** Christopher Heath


### ARTICLE XI.

### *Removal oj Large Fibro-Cystic Tumor of Lower Jaw; Recovery.*

BY CHRISTOPHER HEATH.

For the notes of this case we are indebted to Mr. S. Coupland, ward clerk.

The patient is a native of Cumberland, and has been a carter ever since he was eight years old. His parents are living and healthy. When a child he suffered from suppurating glands in the necks and submaxillary region, which were opened; otherwise he has always enjoyed good health. Never had syphilis. Has lived well; drinks beer freely; is often drunk—i. e., on an average once a week. Has followed his occupation up to the time of his admission. About six years ago he first noticed a small, hard swelling, about the size of a marble, situated in the right cheek, attached to the gum and lower jaw, but moveable under his fingers. From the first it had an aching pain, dull and constant. The tumour continued to increase in size, and four years ago it was lanced in the gum; it then began to discharge, and has continued to do so slightly ever since. He has noticed that the discharge is more abundant after drinking much. It has been lanced twice since, and within the last six months it has been twice tapped by Mr. Watson, of Lancaster: the first tapping just before Christmas; the second, five weeks before admission. Mr. Watson says that at each tapping about four ounces of fluid were drawn off. From the displacement inwards of the two anterior molars and the second bicuspid tooth by the increased growth of the mass, these teeth were extracted two years ago. Since Christmas the tumour has grown with increased rapidity, and has become more tender. During this time the patient says he has lost a stone and a half in weight, and that his appetite has diminished. The patient cannot assign any cause for the origin of the growth. He has had no bad teeth in that jaw. Remember having had a blow on the jaw with a pitchfork handle before the tumour appeared.

July 10.—The patient is fairly built, florid, and sunburnt. He does not look ill. Tongue clean; appetite better than it has been; pulse 76, full and bounding.

The right side of the face presents a large, smooth, globular swelling, which occupies the whole side. It extends in front to the angle of the mouth; behind, to a distance of about an inch behind the lobule of the ear, measuring in this diameter 7½ inches. Above, it extends from the tragus of the ear along the lower margin of the orbit to the side of the nose; below, on a level with the hyoid bone. It measures from above, down over the greatest prominence, 8½ inches. The circumference of the mass measures 18 inches. The right angle of the mouth is drawn slightly upwards and outwards; but the contour of the lower lip is unaffected and the contour of the chin quite preserved. The upper part of the mass is more vascular in appearance than the lower. On the under surface are some cicatrices from the abscesses which were opened when he was a child. The rest of the surface of the tumor is quite smooth, not ulcerated. Temperature of the cheek, 99·7°. The tumor is more tender posteriorly than elsewhere. Its lower two-thirds feel hard and resisting, the skin being quite moveable over the mass. The posterior portion of the tumor is also solid, as well as a portion which extends in front of the ear for about an inch. The rest of the mass is soft and fluctuating, evidently containing fluid, and the upper margin of the solid portion can be distinctly felt across the tumor. Inside the mouth the alveolar border of the right side of the lower jaw is much widened, extending inwards, so as to diminish the cavity of the mouth behind the first bicuspid. The second bicuspid and first and second molar teeth are wanting. The patient says the third molar is present; but it is not visible, nor can it be felt. On the widened alveolar border is an ulcerated surface, covered with a layer of thin purulent fluid, which is continually oozing. The tumor evidently arises from this part of the lower jaw; for, anteriorly, a thin shell of bone can be felt continuous with the jaw and with the surface of the tumor. The upper jaw does not seem to be implicated in the growth. The teeth in it are all present; but the alveolar border has been displaced inwards from the growth of the tumor, so that the roof of the mouth appears contracted.

July 11th.—Patient feels rather sick this morning. Temperature 98·4°; pulse regular, 72.

12th.—10 A.M.: Temperature 99°; pulse 80. Urine, 82 oz.; specific gravity 1013; acid; no albumen.

To-day (12th), at 2 P.M., chloroform having been administered, Mr. Heath proceeded to remove the tumour. He first extracted the right canine and second incisor teeth of the lower jaw, a piece of the jaw coming away with the teeth. He then made a vertical incision to the right of the symphysis through the lip down to the base of the jaw; from the lower end of this incision he cut upwards and backwards over the tumor towards the ear as far as one inch above the angle of the jaw; in making this cut he divided the facial artery. Ligatures were applied before the incision was completed. The whole length of the incision was about nine inches. The superficial tissues were then dissected off the tumor, the large upper flap being first raised; and the tumor was carefully shelled out. In dissecting up this flap the facial artery was again cut and ligatured. The cyst at the upper part of the tumor being now fully exposed, it was laid open by a free incision extending right across it, and and about ten ounces of fluid escaped. Mr. Heath then continued to separate the mass from the skin at the lower part, and, having cleared it as far as the anterior incision, he sawed through the jaw where the teeth had been extracted. He then cut through the mucous membrane and muscles attached to the jaw, and here again some vessels had to be secured. Having completely separated the mass, he attempted to forcibly depress the jaw so as to disarticulate it; but the coronoid process becoming caught against the malar bone, he had to detach the process by the bone forceps. On depressing the jaw, he found that a small portion of the condyle was free from the growth. As he was proceeding to disarticulate, the remains of the lower jaw gave way just below the condyle, the tumor shelling out from the expanded bone around it. The posterior part of the jaw was left nearly down to the angle; a small piece of this was afterwards cut off with the bone forceps. About four ligatures were applied to bleeding vessels, and the rest of the hæmorrhage was arrested by the actual cautery. The wound was then thoroughly sponged out and sewn up; for the incision through the lip hare-lip sutures were employed, and a very fine suture for the mucous membrane of the lip, the rest of the incision being closed by silver wire sutures; the whole of the wound was then painted with collodion. There was not very much blood lost during the operation. The patient was not thoroughly under the influence of chloroform the greater part of the time.

After removal, the tumor was almost globular in form. It measured 3½ inches in diameter at its widest point. It was slightly lobular on the surface. It weighed 13½ ounces. Over the whole of the lower part was a thin shell of expanded bone; for the greater part this was over a solid growth, but the upper and outer aspect was a large cyst capable of containing about 6 ounces of fluid. The lower part of the wall of the cyst was bony, but the whole of the upper part was free from bone. The whole of the inner wall of the cyst was formed of a thin layer of bone. Just anterior to this large cyst was a smaller one containing about $\frac{1}{2}$ ounce of thick fluid, in which was a large quantity of cholesterine. Its walls were bony everywhere. Both cysts were lined by a smooth thin membrane. On the inner side of the tumor were two openings about $\frac{3}{4}$ inch in diameter, which had opened into the mouth. They communicated with a large cavity in the centre of the tumor, into which the finger could be pushed as far as the second joint. On making a section right through the mass, this central cavity was found to be about 2 inches in length. The inner surface was very irregularly lobulated. The lobules varied in size from a pea to a filbert. They were covered by a smooth membrane. The tumor was moderately firm, of a whitish color, and small points of bone were scattered through it. On scraping, it yielded a whitish fluid mixed with fragments of the substance of the tumor. Under the microscope this was found to consist of a few spindle cells and a vast number of free oval nuclei, containing one, two, or three shining nucleoli. Some of the nuclei were perfectly circular. The average diameter was about $\frac{1}{1500}$ inch.

On examining sections made from one of the lobules from the central cavity of the tumor, it was found to consist chiefly of a dense fibrous tissue, amongst which were oval and irregularly shaped spaces, having an appearance much resembling acini and ducts of glands. They were completely filled with oval nuclei, each containing one or more bright shining nucleoli. They were arranged along the walls of the spaces so as to look like epithelium, but they had not the distinct cell and nucleus characteristic of epithelium. The relative proportion of the spaces and fibrous tissue varied greatly. At some parts it was firmly fibrous, and at others the spaces formed the greater part of the growth. The patient made a quick and uninterrupted recovery.—*London Lancet.*

